# Murrah and Sunn herbs induced liver failure

**DOI:** 10.4103/0256-4947.60527

**Published:** 2010

**Authors:** Ibrahim Altraif, Mutasim Dafalla

**Affiliations:** From the Department of Hepatobiliary Sciences and Liver Transplantation, Hepatology Section, King Abdulaziz Medical City, Riyadh, Saudi Arabia

**To the Editor:** Our patient was a 69-year-old Saudi female with diabetes mellitus complicated by peripheral arterial disease, which resulted in an above knee amputation of her left lower limb one year ago. She complained of pus discharge from a sinus at the stump. A course of oral cefalexin was prescribed for her two months prior to presentation, with no benefit. She was only taking insulin for diabetes.

**Figure 1:**
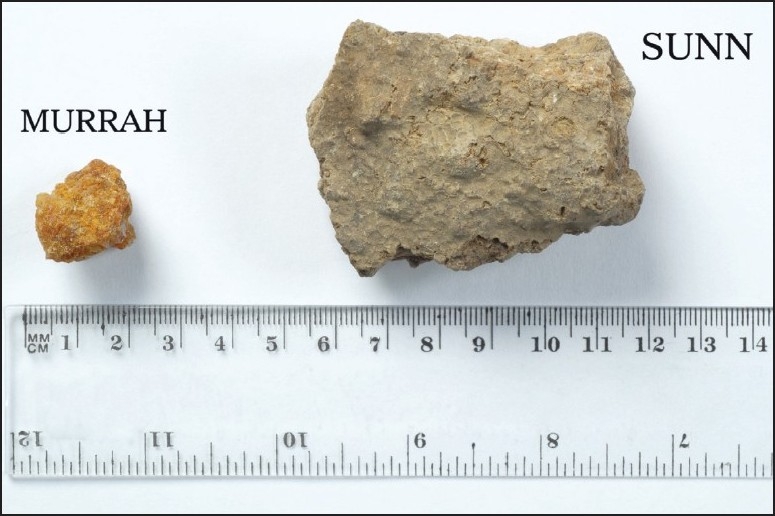
Murrah and Sunn brought by the patient.

The patient was using herbal medications; Murrah and Sunn (composed of the feces of wild rabbits and other unknown components) for the last two months (Figure 1). She used to dissolve 3-5 g of Murrah, in 1-2 L of water with 8-10 g of Sunn. After simple filtration, about 200 mL of the solution was taken orally three to five times a day, continuously, for two months.

She presented to King Abdulaziz Medical City with complaints of nausea, vomiting, fatigue, and right hypochondriac abdominal pain. On examination, she was found to have normal vital signs with mild dehydration. There were no stigmata of chronic liver disease or encephalopathy, and she previously had a completely normal liver biochemistry. Initial laboratory results revealed leukocytosis and transaminitis with increased INR 4.3 (Table 1). HBs Ag, HAV IgM, and HCV-RNA were negative, as well as serology for Herpes virus, Cytomegalovirus (CMV), Epstein-Barr virus (EBV), Legionella, and mycoplasma. Autoimmune markers; ANA, ASMA, and AMA and metabolic screens: s.ceruloplasmin, alpha_1_ antitrypsin, and iron studies were all normal.

**Table 1:** Laboratory tests.

	Day 1	Day 2	Day 3	Day 8	Day 20	Day 35
Bilirubin (μmol/L)	27	20	67	33	18	16
ALT/AST (U/L)	942/913	>4700/>4500	2468/972	604/96	67/40	20/26
ALK-P/ GTP (U/L)	548/168	476/173	471/179	323/122	199/92	186/80
International normalized ratio	4.3	4.2	2.2	1.3	1.2	1.1
Albumin (G/L)	36	30	27	29	31	32
WBC/HB/PLT	21.7/11.6/130	17.5/11.5/115	15.5/12.0/132	5.3/12.0/175	3.6/11.9/196	7.2/12.7/274

ALT: alanine aminotransferase, AST: asportate aminotransferase, ALK-P: alkaline phosphatase, WBC: white blood cells, Hb: hemoglobin, PLT: platelets

**Figure 2:**
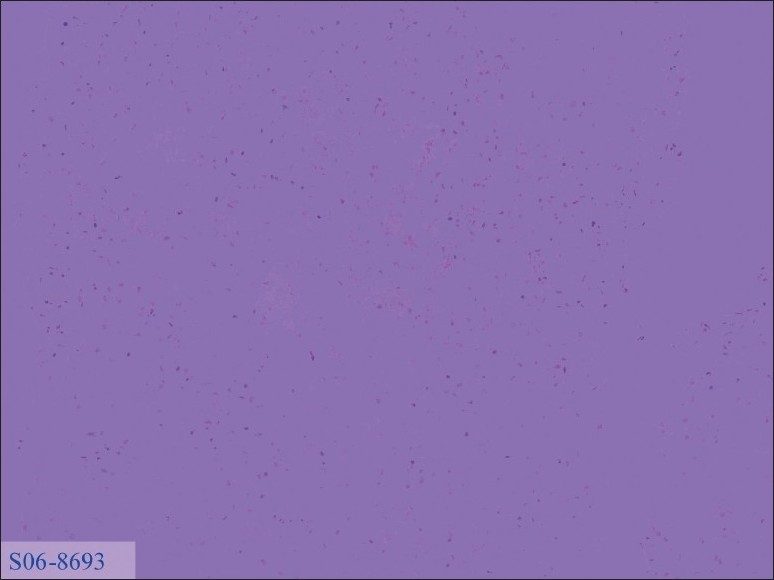
Prominent hepatocyte necrosis (> 60%) predominantly involving zone 3, associated with marked congestion of the perivenularhepatic sinusoids

**Figure 3:**
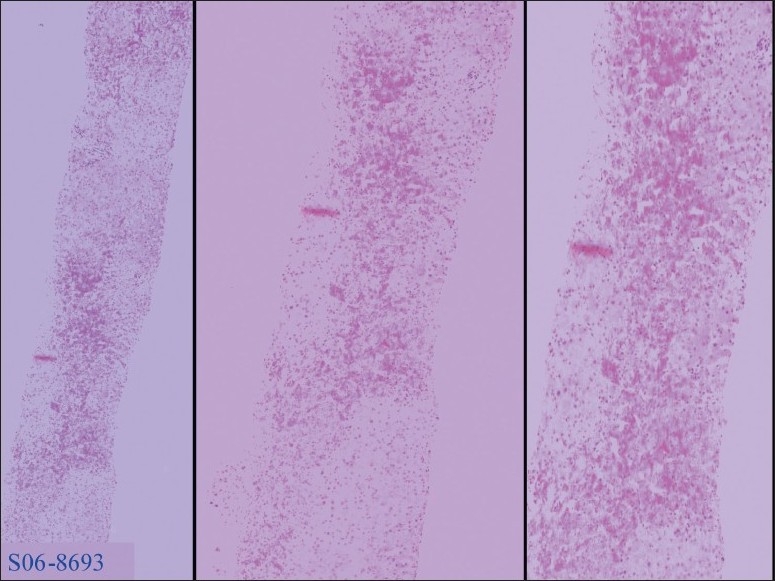
Higher magnification showing hepatocyte necrosis and marked congestion of the perivenularhepatic sinusoids. The central veins and portal tracts are unremarkable.

Renal function, electrocardiogram (EKG), and echocardiography were unremarkable. Transjugular liver biopsy revealed prominent hepatocyte necrosis, >60%, predominantly involved zone 3, associated with marked congestion of the perivenularhepatic sinusoids (Figure 2). The central vein and portal tracts were unremarkable. Occasional eosinophilic bodies were noted in the uninvolved hepatic parenchyma (Figure 3).

These histological features were consistent with either drug- or herbal-induced centrilobular hepatic necrosis.

The patient was rehydrated and was placed on insulin, intravenous Vitamin K, and cefuroxime (after wound swab culture), and was asked to stop taking herbs.

The patient showed great improvement, clinically and biochemically over the following days.

The toxicology report from King Faisal Specialist Hospital and Research Center and the analysis of the samples, concluded that only sample No. 2, Sunn, was not suitable for human use, as it contained toxic quantities of lead and arsenic in addition to P-cresol, which is known to cause hepatotoxcity.^1^,^2^

The patient did not have ischemic hepatitis, as she was having only mild dehydration and maintained her blood pressure, with a normal EKG, unremarkable echocardiography, and normal renal function. Furthermore, she was taking only insulin and previously had a normal liver biochemistry, excluding the possibility of either chronic liver injury or other drug-induced hepatitis.

Herbal hepatotoxicity typically presents after several weeks or months of continuous herbal use,^3^–^6^ such as in the case of our patient. People use Murrah for a few days without significant problems or perhaps develop subclinical hepatitis, which goes unrecognized. However, hepatotoxic injury varies from focal to extensive hepatocyte necrosis, chronic hepatitis, steatosis, cirrhosis, and veno-occlusive disease.^7^

We believe, that our patient was taking these herbs in large doses for a long period, which led to hepatotoxicity and liver injury, as a result of contamination and accumulation of these toxic heavy metals and p-cresol, which was one of the organic compounds that was categorized as a phenol (sometimes called phenolics). Depending on the temperature, cresols could be solid or liquid because they had melting points not far from room temperature. Similar to other types of phenols, they were oxidized slowly by long exposure to air and the impurities often gave cresols a yellowish to brownish-red tint. Paracresol (p-cresol) was used to dissolve other chemicals, as a disinfectant, deodorizer, and to make specific chemicals as pesticide. It is known to cause multi-organ damage, including liver injury.^6^,^8^

In addition, we could not rule out the possibility of interaction between these herbs, leading to more extensive liver necrosis and damage. We recommend health education addressing the risk of using herbs as medication, and health authorities must maintain and supervise herbal shops with regard to the safety, preparation, storage, and dispensing.
